# Supplementary material for: Supramammillary neurons projecting to the septum regulate dopamine and motivation for environmental interaction in mice
Source: Nat Commun. 2021 May 14;12:2811. doi: 10.1038/s41467-021-23040-z (PMC8121914; doi:10.1038/s41467-021-23040-z)
Supplement: Supplementary file 1 — Supplementary Information [file 41467_2021_23040_MOESM1_ESM.pdf]

# Supplementary Information for

## Supramammillary neurons projecting to the septum regulate dopamine and motivation for environmental interaction in mice

Andrew J. Kesner, Rick Shin, Coleman B. Calva, Reuben F. Don, Sue Junn, Christian T. Potter, Leslie A. Ramsey, Ahmed F. Abou-Elnaga, Christopher G. Cover, Dong V. Wang, Hanbing Lu, Yihong Yang, and Satoshi Ikemoto

Correspondence to: satoshi.ikemoto@nih.gov

This PDF file includes:

Supplementary Figures 1 to 9

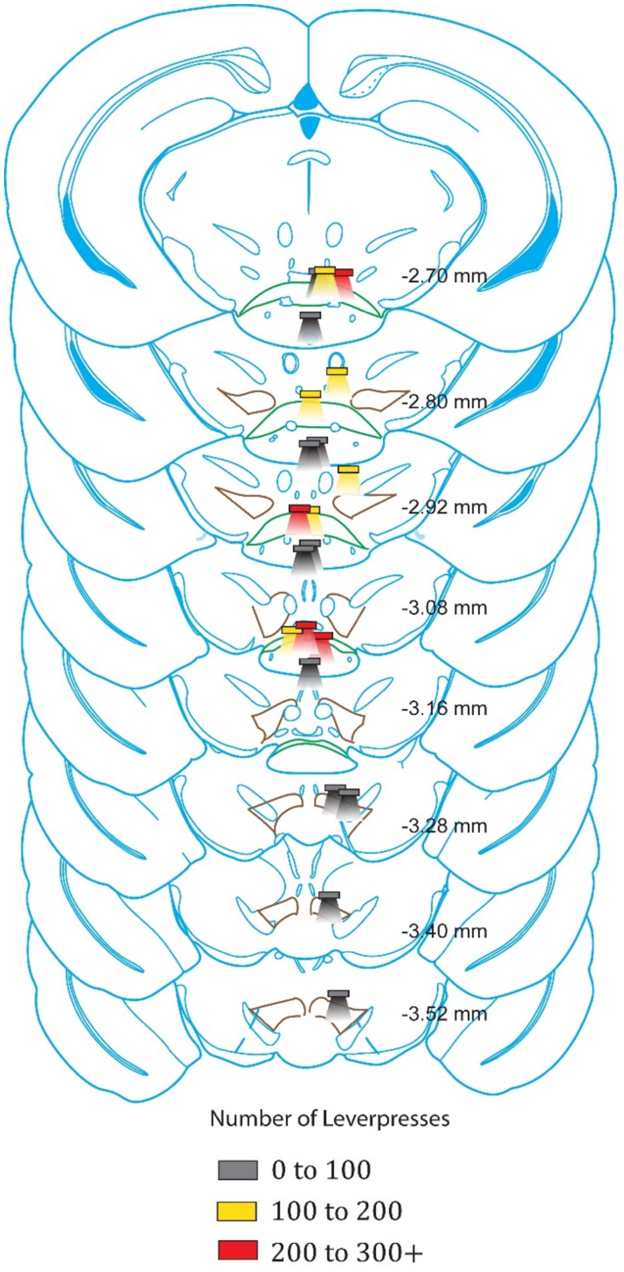


**Supplementary Figure 1, related to Figure 1a-e: Optic fiber placements and effectiveness for SuM subpopulation stimulation**

Diagram of coronal sections indicating placements of optic fibers and relative active lever-press levels at that location. Rectangles indicate tips of optic fiber and below is approximate area affected by illumination. Colors indicate levels of active lever presses in 30-minute session; see key in figure; values are calculated from average lever presses from last three acquisition sessions. The SuM is outlined in green.

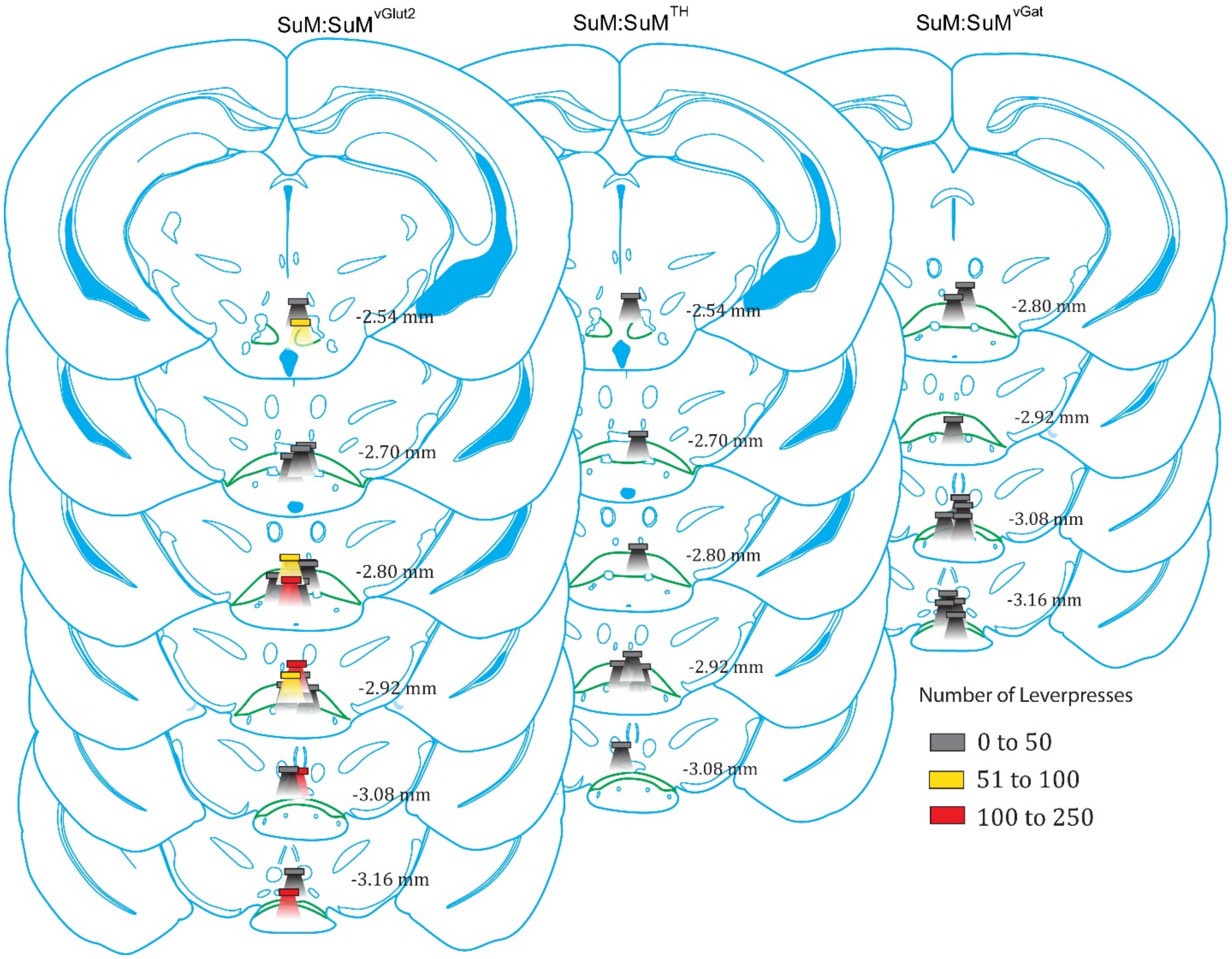


**Supplementary Figure 2, related to Figure 1f-h: Optic fiber placements and effectiveness for SuM glutamatergic neuron terminal stimulation**

Diagram of coronal sections indicating placements of optic fibers and relative active lever-press levels at each location. Rectangles indicate tips of optic fiber and below is approximate area affected by illumination. Colors indicate levels of active lever presses in 30-minute session; see key in figure; values are calculated from average lever presses from last three acquisition sessions. The SuM is outlined in green.

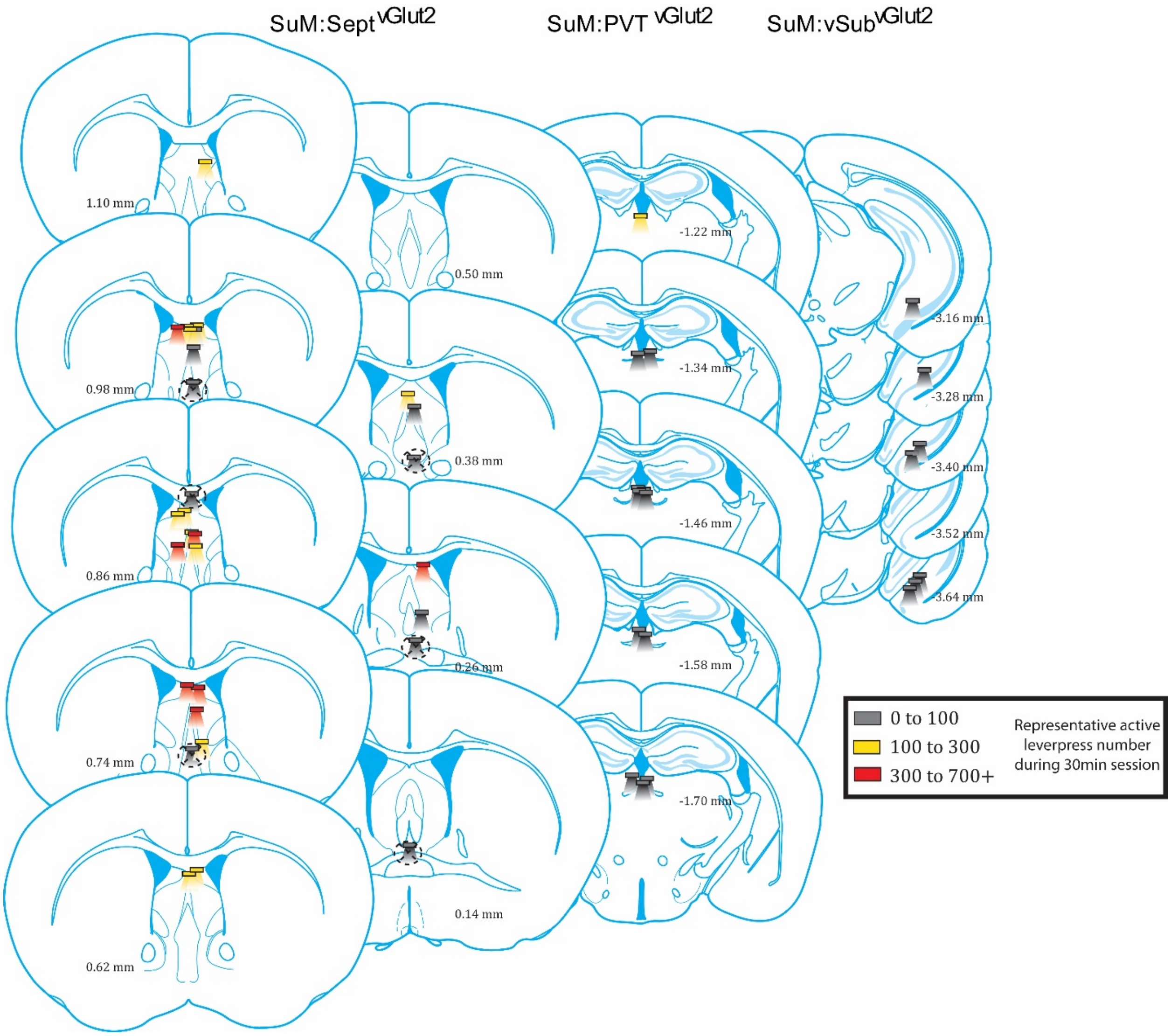


**Supplementary Figure 3, related to Figure 2: Optic fiber placements and effectiveness for terminal stimulation**

Diagram of coronal sections indicating placements of optic fibers and relative active-lever press levels at each location. Rectangles indicate tips of optic fiber and below is approximate area affected by illumination. Colors indicate levels of active lever presses in 30-minute session; see key in figure; values are calculated from average lever presses from last three acquisition sessions. Mice with septal area fiber placements in the corpus collosum or past the level of anterior commissure did not lever press and were removed from analysis (marked by dotted circle in figure; n=6).

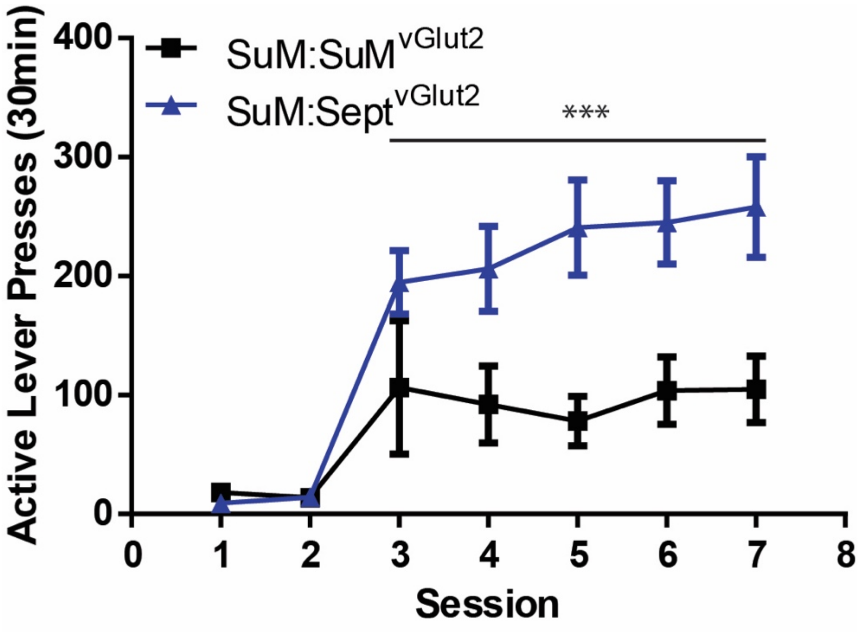


**Supplementary Figure 4, related to Figure 2: Summary of ICSS responses for SuM glutamate cell body vs septum terminals**

Active lever presses per session were compared between SuM:SuM$^{vGlut2}$ (n=12) and SuM:Sept$^{vGlut2}$ (n=23) (means $\pm$ SEM). Terminal stimulation supported greater lever press rates during sessions 3-7 ($2_{group}$ x $5_{session}$ ANOVA; $F_{group}(1,22)=7.619$, ***p=0.009). Source data are provided as a Source Data file.

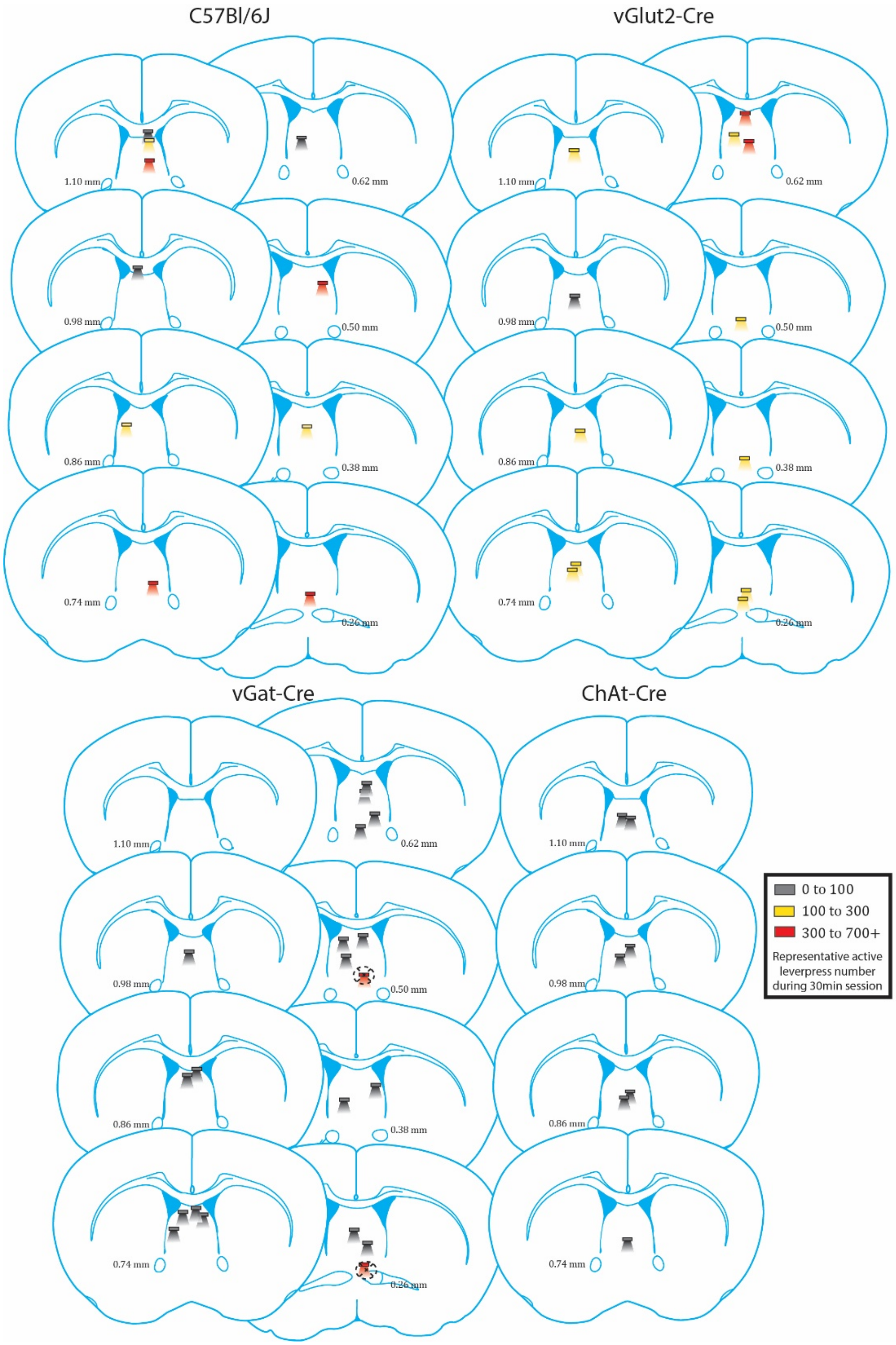


**Supplementary Figure 5, related to Figure 4: Optic fiber placements and effectiveness for terminal stimulation**

Diagram of coronal sections indicating placements of optic fibers and relative active-lever press levels at each location. Rectangles indicate tips of optic fiber and below is approximate area affected by illumination. Colors indicate levels of active lever presses in 30-minute session; see key in figure; values are calculated from average lever presses from last three acquisition

sessions. vGat-Cre mice with fiber placements very ventral to the septum, and near nucleus accumbens or bed nucleus of stria terminalis lever pressed at extremely high rates, likely due to stimulation of these known reward nuclei and were removed from analysis (marked by dotted circle in figure; n=2)

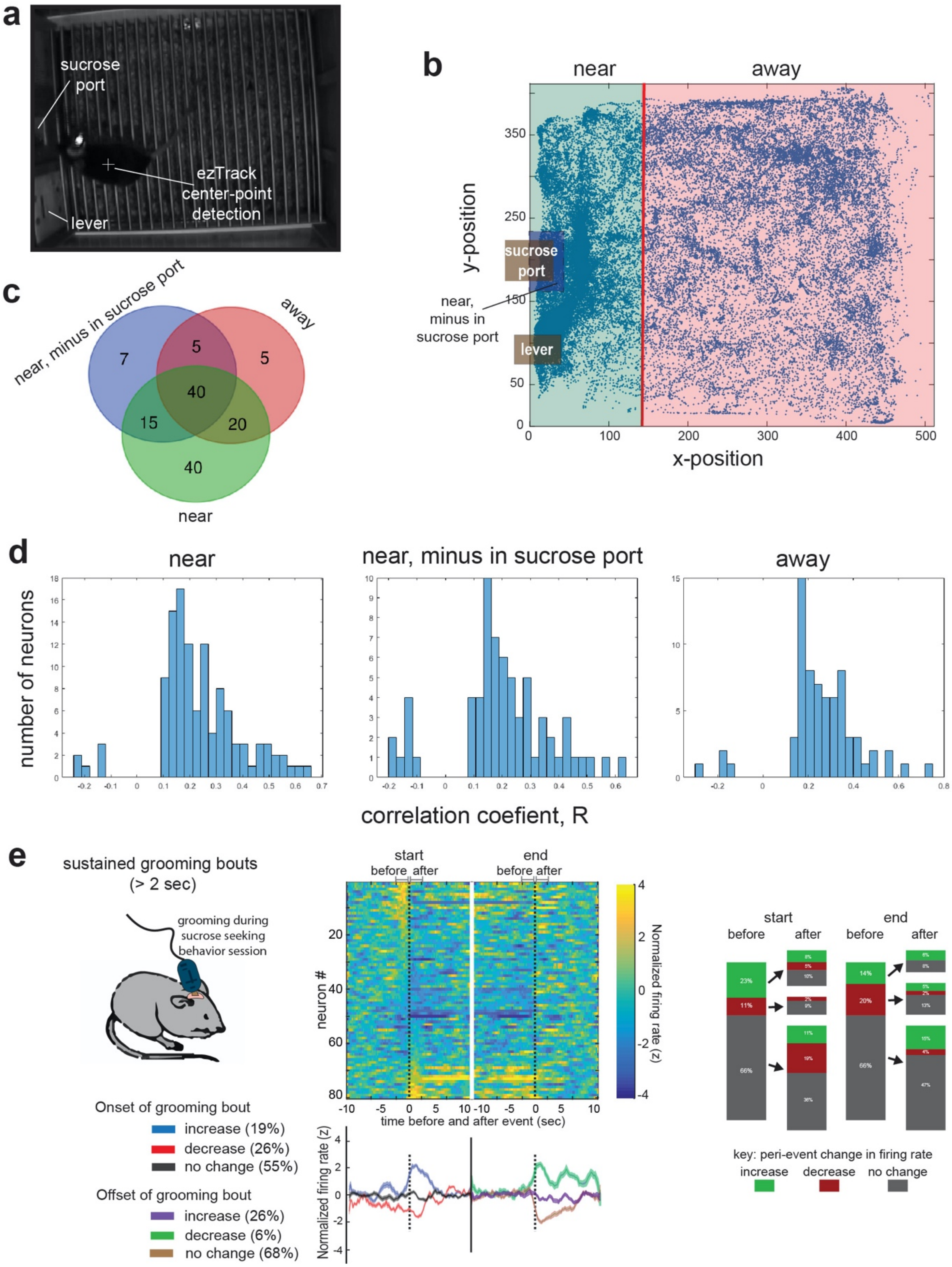


**Supplementary Figure 6, related to Figure 5: Categorization of SuM neurons while mice locomote or groom during seeking behavior recordings**

**a** Example snap-shot image from a video used to track a mouse during single-unit recording sessions. Sucrose solution reward port, retracted lever, and software determined center point of animal are labeled.

**b** X and Y coordinate plot of every center point measurement of an example video tracking analysis. The mean X-coordinate position is marked with a vertical red line (mean x-pos). Time points where the center point is to the left (or less-than) this position on the x-axis were categorized as “near” and the opposite for “away”. Notice the density of points are skewed towards the sucrose port and lever side of the chamber, and as such the mean x-pos is shifted towards that side as well.

**c** Venn diagram of single-units (132/152) that correlate to locomotor activity during epochs when the mouse is ‘away’ (red), ‘near’ (green), or near but not inside the sucrose port (blue), and whether correlations exist across different epochs.

**d** Histogram plots of the range in R-value correlation coefficients for single-units who’s firing rate significantly correlated to locomotor activity when mice are ‘near’ (left), ‘near’ but not inside sucrose port (middle), and ‘away’ from sucrose port.

**e** Time stamps corresponding to grooming (cartoon on left) bout starts and ends were scored visually offline by a trained researcher using video recordings of the behavior sessions. Only bouts greater than 2 seconds in length were used for analysis. Some animals did not have such bouts during a few sessions, so number of neurons recorded is less in this analysis since some sessions were discarded. Middle-Top: Heatmaps of normalized firing rates of SuM neurons during start and end of grooming bouts. Middle-Bottom: Traces indicate mean normalized firing rate (+ SEM) of neurons that either increase, decrease or show no change in firing rate in response to beginning and ending of grooming bouts. Plotted on the same time scale as the raster plot above. The percentage of neurons in each category is shown in the boxes below. Left: Changes in firing rate of neurons before and after grooming starts and ends. Source data are provided as a Source Data file.

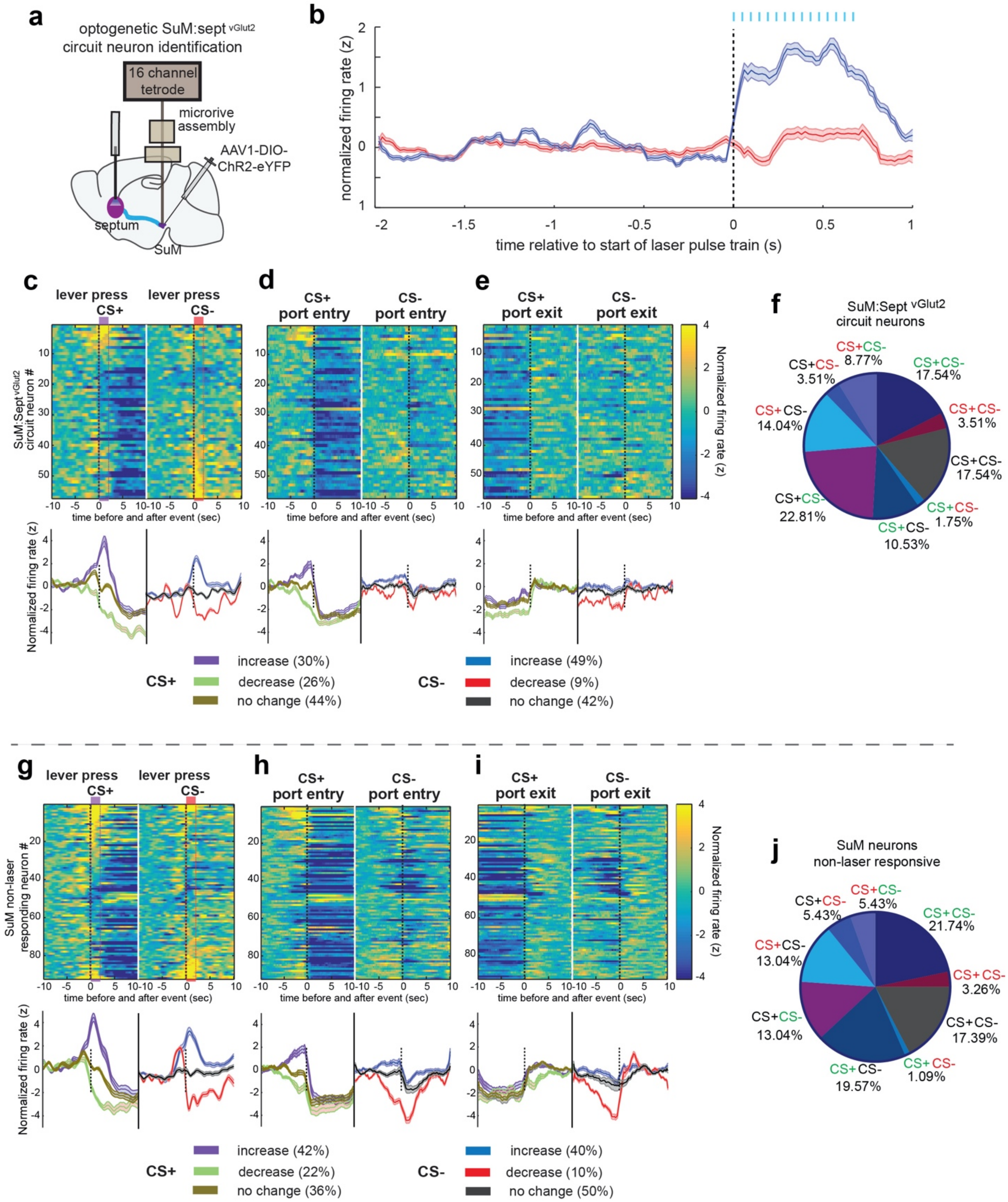


**Supplementary Figure 7, related to Figure 5: Similar neural activities between SuM neurons that were confirmed to project to the septum and those were not**

**a** Schematic showing the preparation of SuM:Sept$^{vGlut2}$ optogenetic stimulation and microdrive tetrode recordings in SuM.

**b** Traces (mean ± SEM) of neurons categorized as SuM:Sept$^{vGlut2}$ circuit neurons (blue) and neurons that did not respond to laser pulse train, i.e. SuM non-laser responding neurons (red). We found that 37.5% (n=57/152) of SuM neurons significantly increased firing rate to laser stimulation (z-score normalized to a baseline period 2 s before laser pulse train was >= 1) and were further analyzed as SuM:Sept$^{vGlut2}$ circuit neurons. Additionally, 60.5% (n=92/152) of SuM neurons showed no change in firing rate as a function of laser stimulation (z-score absolute value < 1). It should be noted that some of non-responsive neurons most likely projected to the septum, since photostimulation must have recruited only a fraction of SuM neurons projecting to the septum. Finally, 2% (n=3/152) of the neurons showed decreased firing rate but were not further analyzed.

**c-e** Heatmaps of normalized firing rates of SuM:Sept$^{vGlut2}$ circuit neurons during lever presses that initiate CS+ or CS- events (c), and nose poke entry (d) and nose poke withdrawing (e) that follow CS+ or CS- lever presses. The neuron order is constant across the heatmaps and are based off cue responsiveness in (a). Below heatmaps are respective traces indicating mean normalized firing rate (± SEM) of neurons that are categorized as increasing, decreasing, or non-responding to CS+ and CS- with the same time scale as the raster plot above. The percentage of neurons in each category is shown in the boxes below.

**f** Proportions of neurons that are categorized as SuM:Sept$^{vGlut2}$ circuit neurons responding to CS+ and CS- cues in particular manner (firing rate increase = green, decrease = red, no response to cue = black).

**g-j** Same analysis as in c-f, but for SuM neurons that showed no change in firing rate as a function of SuM:Sept$^{vGlut2}$ stimulation. Source data are provided as a Source Data file.

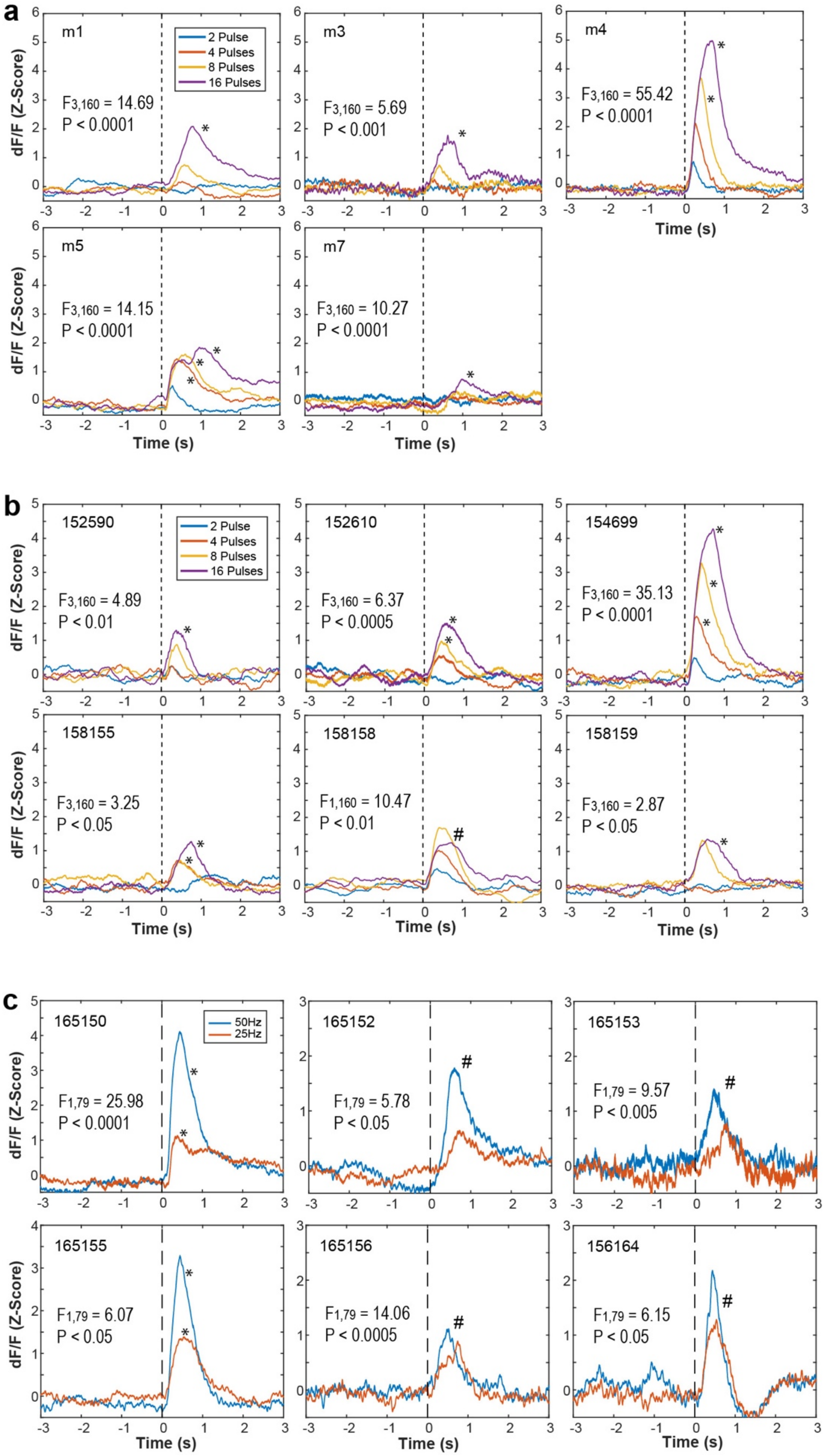
a
m1
m3
m4
m5
m7
2 Pulse
4 Pulses
8 Pulses
16 Pulses
F3,160 = 14.69
P < 0.0001
F3,160 = 5.69
P < 0.001
F3,160 = 55.42
P < 0.0001
F3,160 = 14.15
P < 0.0001
F3,160 = 10.27
P < 0.0001
dF/F (Z-Score)
Time (s)
b
152590
152610
154699
158155
158158
158159
F3,160 = 4.89
P < 0.01
F3,160 = 6.37
P < 0.0005
F3,160 = 35.13
P < 0.0001
F3,160 = 3.25
P < 0.05
F1,160 = 10.47
P < 0.01
F3,160 = 2.87
P < 0.05
c
165150
165152
165153
165155
165156
156164
50Hz
25Hz
F1,79 = 25.98
P < 0.0001
F1,79 = 5.78
P < 0.05
F1,79 = 9.57
P < 0.005
F1,79 = 6.07
P < 0.05
F1,79 = 14.06
P < 0.0005
F1,79 = 6.15
P < 0.05

**Supplementary Figure 8. Results of fiber-photometry $Ca^{+2}$-imaging experiments**

**a-c** The number on the upper left corner of each plot indicates subject ID. (a) VTA GCaMP signals (Z-score dF/F) of VTA DA neurons as a function of stimulation (25 Hz; 2, 4, 8 and 16 pulses) delivered at the SuM-to-septum pathway. (b) VStr dLight signals as a function of stimulation (25 Hz; 2, 4, 8 and 16 pulses) delivered at MS GLU neurons. (c) VStr dLight signals as a function of stimulation (16 pulses; 25 and 50 Hz) delivered at MS-to-VTA GLU neurons. The F- and P-values shown within each graph indicate the interaction of a repeated ANOVA with Time (before and after stimulation train) and stimulation parameter on areas under curve. When the interaction was significant, multiple comparison t-tests with Benjamini and Hochberg correction were conducted on Time for each pulse. * $P < 0.05$. When the interaction was not significant, F and P-values of main Time effect is shown. $^{\#}P < 0.05$, with stimulation parameters combined.

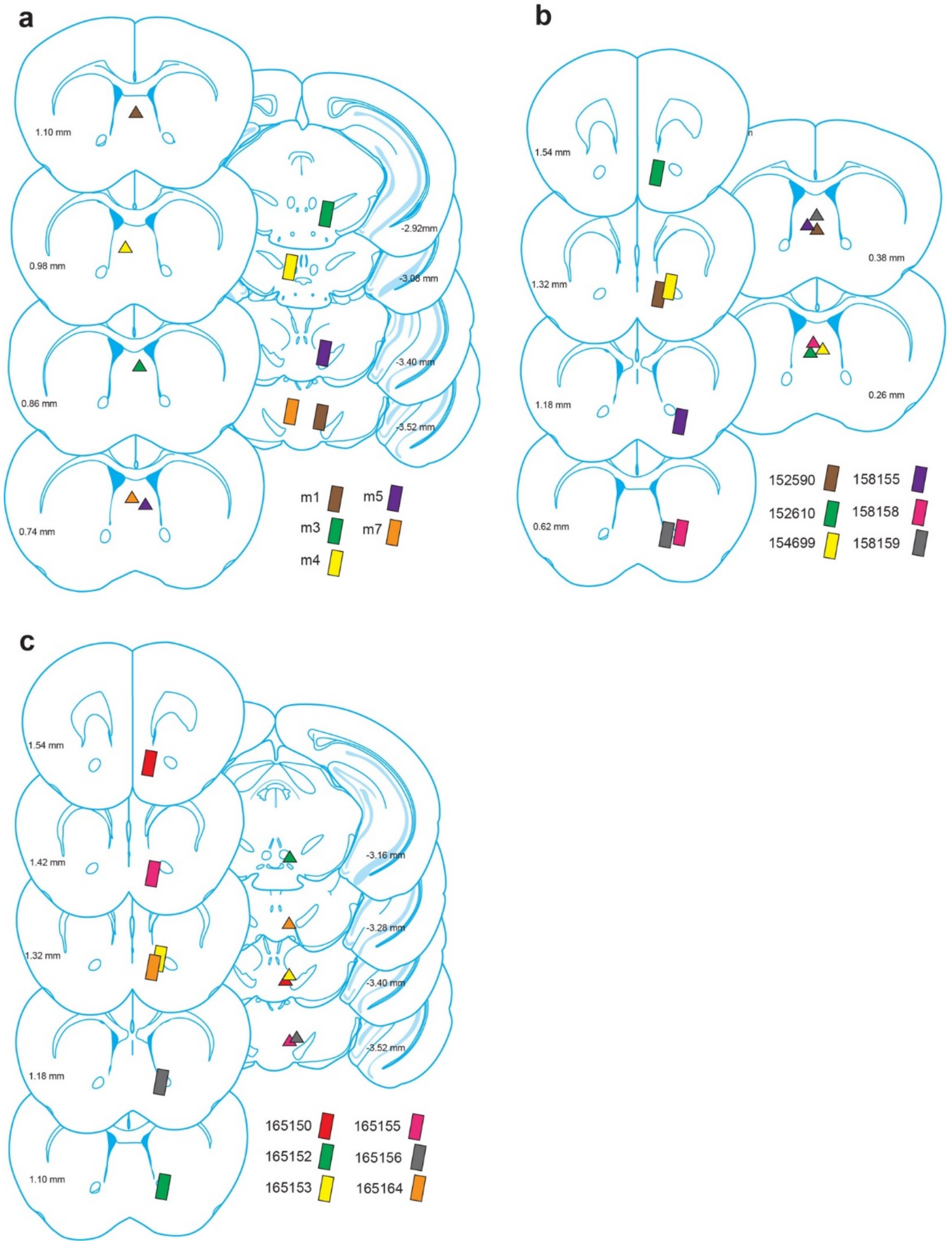


**Supplementary Figure 9. Fiber-photometry Ca2+ signal data showing the activation of the VTA-VStr DA system.**

**a-c** Diagram of coronal sections indicating placements of optic fibers used for optogenetic stimulation (triangles) and fiber photometry fibers for photometric recording (rectangles), used for experiments testing SuM-MS GLU stimulation on VTA DA neuron activity (a), and MS GLU (b) or MS-VTA GLU (c) neuron stimulation on DA release in VStr. The respective placements for each mouse of each experiment are color matched.
