# Supplementary material for: Supramammillary neurons projecting to the septum regulate dopamine and motivation for environmental interaction in mice
Source: Nat Commun. 2021 May 14;12:2811. doi: 10.1038/s41467-021-23040-z (PMC8121914; doi:10.1038/s41467-021-23040-z)
Supplement: Supplementary file 3 — Reporting Summary [file 41467_2021_23040_MOESM3_ESM.pdf]

nature research

Corresponding author(s): Dr. Satoshi Ikemoto

Last updated by author(s): Jan 31, 2021

Reporting Summary

Nature Research wishes to improve the reproducibility of the work that we publish. This form provides structure for consistency and transparency in reporting. For further information on Nature Research policies, see our Editorial Policies and the Editorial Policy Checklist.

Statistics

For all statistical analyses, confirm that the following items are present in the figure legend, table legend, main text, or Methods section.

n/a	Confirmed	
☐	☒	The exact sample size (*n*) for each experimental group/condition, given as a discrete number and unit of measurement
☐	☒	A statement on whether measurements were taken from distinct samples or whether the same sample was measured repeatedly
☐	☒	The statistical test(s) used AND whether they are one- or two-sided<br>*Only common tests should be described solely by name; describe more complex techniques in the Methods section.*
☐	☒	A description of all covariates tested
☐	☒	A description of any assumptions or corrections, such as tests of normality and adjustment for multiple comparisons
☐	☒	A full description of the statistical parameters including central tendency (e.g. means) or other basic estimates (e.g. regression coefficient) AND variation (e.g. standard deviation) or associated estimates of uncertainty (e.g. confidence intervals)
☐	☒	For null hypothesis testing, the test statistic (e.g. *F*, *t*, *r*) with confidence intervals, effect sizes, degrees of freedom and *P* value noted<br>*Give P values as exact values whenever suitable.*
☒	☐	For Bayesian analysis, information on the choice of priors and Markov chain Monte Carlo settings
☒	☐	For hierarchical and complex designs, identification of the appropriate level for tests and full reporting of outcomes
☐	☒	Estimates of effect sizes (e.g. Cohen's *d*, Pearson's *r*), indicating how they were calculated

Our web collection on statistics for biologists contains articles on many of the points above.

Software and code

Policy information about availability of computer code

Data collection	We used commercial software for data collection, including: 1) MED-PC IV; Med-associates. 2) Neuralynx Digital Lynx data acquisition system running Neuralynx Cheetah software. 3) Nodlus, Ethovision. 4) WinWCP. 5) Doric Neuroscience Studio. 6) Bruker Biospin 9.4T scanner
Data analysis	We used commercial software for data analysis, including: 1) ImageJ v1.5; NIH. 2) Matlab (2016b); Mathworks. 3) Prism v7; GraphPad. 4) Statistica; StatSoft. 5) Offline Sorter V3; Plexon. 6) Neuroexplorer v4; Nex Technologies. 7) AxoGraph X. 8) AFNI (ver: AFNI_18.3.10, https://afni.nimh.nih.gov/) 9) ANTs (ver: 2.2.0, http://stnava.github.io/ANTs/). 10) SPM12 (ver: v7771, https://www.fil.ion.ucl.ac.uk/spm).<br>And open source software; 1) EzTrack v1.0 (https://github.com/DeniseCaiLab/ezTrack).

For manuscripts utilizing custom algorithms or software that are central to the research but not yet described in published literature, software must be made available to editors and reviewers. We strongly encourage code deposition in a community repository (e.g. GitHub). See the Nature Research guidelines for submitting code & software for further information.

Data

Policy information about availability of data

All manuscripts must include a data availability statement. This statement should provide the following information, where applicable:

- Accession codes, unique identifiers, or web links for publicly available datasets
- A list of figures that have associated raw data
- A description of any restrictions on data availability

The data that support the findings of this study are available from the authors on reasonable request.

Field-specific reporting

Please select the one below that is the best fit for your research. If you are not sure, read the appropriate sections before making your selection.

☒ Life sciences ☐ Behavioural & social sciences ☐ Ecological, evolutionary & environmental sciences

For a reference copy of the document with all sections, see nature.com/documents/nr-reporting-summary-flat.pdf

Life sciences study design

All studies must disclose on these points even when the disclosure is negative.

Sample size	All sample sizes for experiments were determined based on our prior experience performing similar experiments. For example, for in-vivo studies involving brain implants (e.g. optogenetics or microcannulations), it impossible to target the same exact spot in every mouse, so typically more mice were prepared and run in experiments than the 5-10 generally needed for appropriate statistical comparisons in behavioral neuroscience studies, with the expectation that several will be removed upon histology. Thus all behavioral data was analyzed after histological examination of brain tissue. For examples of our prior publications using similar techniques, see: for assessing brain stimulation reward or aversion - PMIDs 24431440, 24834037; small rodent fMRI - PMID 33446857; slice-physiology experiments - PMID 29024664. For in-vivo electrophysiology experiments the sample sizes were chosen based off of the number of neurons successfully being recorded from each mouse until total number of neurons collected were considered sufficient to conduct statistical analyses based on our previous experience and published research (see PMID 28297663, 25867120).
Data exclusions	We excluded animals after posthoc histological analysis indicated placements of neural probes were outside the regions being studied. In addition, some animal were excluded when they failed to display spontaneous lever pressing, which made assessing conditioned operant behavior rewarded by brain stimulation impossible.
Replication	Most of our data was collected in several cohorts of mice due to the time and space limitations of conducting behavioral research. Typically each experiment required 2-3 cohorts of mice independently prepared, and thus our behavioral experiments consisted of several replicates of each experiment. In each cohort, roughly 10-20% of mice were excluded after histological analysis or not meeting predetermined behavioral criteria.
Randomization	Mice were selected at random for all groups with counterbalanced assignments during treatments and when changing experimental conditions (e.g. stimulation site).
Blinding	Blinding is not realistically achievable to our optogenetic experiments, since experimenters are observing animals during behavioral studies, are physically connecting mice to optic fibers targeting specific brain regions, and are able to see optogenetic stimulation occurring. However, mice were not included in study until after histological analysis, which was conducted blinded to the animal's prior behavior.<br>When different drugs or doses are tested, we did so in a random counterbalanced design when possible, or in a predetermined dosing regimen based potential carry over effects.

Reporting for specific materials, systems and methods

We require information from authors about some types of materials, experimental systems and methods used in many studies. Here, indicate whether each material, system or method listed is relevant to your study. If you are not sure if a list item applies to your research, read the appropriate section before selecting a response.

Materials & experimental systems

n/a	Involved in the study	
☐	☒	Antibodies
☒	☐	Eukaryotic cell lines
☒	☐	Palaeontology and archaeology
☐	☒	Animals and other organisms
☒	☐	Human research participants
☒	☐	Clinical data
☒	☐	Dual use research of concern

Methods

n/a	Involved in the study	
☒	☐	ChIP-seq
☒	☐	Flow cytometry
☐	☒	MRI-based neuroimaging

Antibodies

Antibodies used	rabbit anti-cFos; Santa Cruz Biotechnology; Cat# sc-52<br>goat anti-GFP; abcam; Cat# ab5450<br>donkey anti-goat Alexa Flour 488; Life Technologies; Cat# A-11055<br>donkey anti-rabbit Alexa Flour 594; Life Technologies; Cat# A-11012
Validation	These are commonly used antibodies and have a long publication and validation record.

Validation | See website for citation list (n=597) for cFos Ab: https://www.scbt.com/scbt/product/c-fos-antibody-4
See website for citation list (n=105) for GFP Ab: https://www.abcam.com/gfp-antibody-ab5450.html

Animals and other organisms

Policy information about studies involving animals; ARRIVE guidelines recommended for reporting animal research

Laboratory animals

Male and female mice; aged 2-4mo; 25-35g at time of study.
Mouse: (Vglut2)::IRES-Cre (Slc17a6tm2(cre)Lowl); The Jackson Laboratory; Stock# 016963
Mouse: (vGat)::IRES-Cre (Slc32a1tm2(cre)Lowl); The Jackson Laboratory; Stock# 016962
Mouse: ChAT-IRES-Cre::frt-neo-frt (Chattm2(cre)Lowl); The Jackson Laboratory; Stock# 006410
Mouse: C57BL/6J; The Jackson Laboratory; Stock# 000644
Mouse: (Th)::IRES-Cre; Lindeberg et al., 2004; N/A

Rat: Male; Wistar; Envigo; Outbred; 250-300g at time of study.

Wild animals

The study did not involve wild animals

Field-collected samples

The study did not involve samples collected from the field

Ethics oversight

All procedures were approved by the Animal Care and Use Committee of the Intramural Research Program, National Institute on Drug Abuse and were in accordance with the Guide for the care and use of laboratory animals (National Research Council, 2011).

Note that full information on the approval of the study protocol must also be provided in the manuscript.

Magnetic resonance imaging

Experimental design

Design type

Block designed optogenetic stimulus fMRI (opto-fMRI).

Design specifications

Block-design optogenetic stimulation was delivered to the SuM or VStr with a train of 25Hz photo-stimulation. The stimulation paradigm consisted of 20s baseline followed by 5 cycles of 20s stimulus-on and 40s stimulus-off. Laser intensity was set to deliver 10 mW of 437 nm light at the tip of the optic fiber.

Behavioral performance measures

During the scanning, animals were kept anesthetized with a combination of isoflurane (0.5%) and dexmedetomidine hydrochloride (0.015 mg/kg/hr). No behavioral measures were taken. Heart rate and blood oxygenation levels were continuously monitored using a noninvasive pulse oximetry attached to the animal's hind foot, while respiration rate was monitored with a MouseOx sensor (Starr Life Sciences, Oakmont, PA, USA) beneath the animal's chest. Body temperature was maintained at 37.1 ± 0.50C and oxygenation saturation was maintained above 96% during fMRI data acquisition.

Acquisition

Imaging type(s)

Functional and structural images

Field strength

9.4 Tesla

Sequence & imaging parameters

Mouse structural MRI: High-resolution anatomical images were acquired using a Rapid Acquisition with Relaxation Enhancement (RARE) sequence (TE = 35ms, TR = 2200 ms, TE = 35ms, FOV = 30×30 mm2, slice thickness = 0.6 mm, slice number = 25).

Mouse fMRI: FMRI data were acquired using a T2*-weighted single-shot EPI sequence (TE = 15 ms, TR = 1000 ms, segment = 2, FOV = 25 × 15 mm2, matrix size = 96×58, slice thickness = 0.6 mm, slice number = 15).

Area of acquisition

Whole brain

Diffusion MRI ☐ Used ☒ Not used

Preprocessing

Preprocessing software

Preprocessing was performed using AFNI software package. FMRI data were preprocessed with slice timing correction, head motion correction, spatial normalization and smoothing.

Normalization

High-resolution anatomical images were co-registered onto a template using AFNI function 3dAllineate. FMRI time series were then co-registered using the 3dvolreg function in AFNI. EPI voxels were resampled to a larger voxel size (0.375 x 0.375 x 0.6 mm3) and spatially normalized using a full-width-at-half-maximum kernel of 0.5 mm to increase the signal-to-noise ratio (SNR)

Normalization template

A mouse template generated from the current anatomical images of the mice after co-registration.

Noise and artifact removal

6 motion parameters were included in the first level analysis to remove motion induced artifact.

Volume censoring

Censoring was not performed for mouse fMRI data as the animals were kept anesthetized and head motion was minimal during fMRI scanning.

Statistical modeling & inference

Model type and settings

The first-level analysis was conducted using general linear modelling (GLM) implemented with AFNI's command 3Ddeconvovle. Head motion parameters were used as nuance variables in the model.

Effect(s) tested

Group activation maps were derived through a voxel-wise beta-weighted t-test against zero using 3dttest in AFNI. Independent two sample t-tests were conducted to compare brain activation difference between two groups of animals received opto-stimulation at different locations.

Specify type of analysis: ☐ Whole brain ☐ ROI-based ☒ Both

Anatomical location(s)

The supramammillary region and nucleus accumbens was defined using task activation and resting state functional connectivity.

Statistic type for inference
(See Eklund et al. 2016)

One-sample t-tests and independent two-sample t-tests were conducted based on questions to address. FMRI results were corrected for multiple comparisons using randomization and permutation simulation in AFNI's program 3dttest++ to achieve corrected P < 0.01 with a minimum cluster size of 20 voxels.

Correction

FWE, Permutation and simulation

Models & analysis

n/a	Involved in the study
☐	☒ Functional and/or effective connectivity
☒	☐ Graph analysis
☒	☐ Multivariate modeling or predictive analysis

Functional and/or effective connectivity

None.
